# Histone Deacetylases Inhibitors in the Treatment of Retinal Degenerative Diseases: Overview and Perspectives

**DOI:** 10.1155/2015/250812

**Published:** 2015-06-02

**Authors:** Hua Zhang, Xufeng Dai, Yan Qi, Ying He, Wei Du, Ji-jing Pang

**Affiliations:** ^1^Eye Hospital, School of Ophthalmology and Optometry, Wenzhou Medical University, Wenzhou, Zhejiang 325027, China; ^2^Department of Ophthalmology, University of Florida, Gainesville, FL, USA

## Abstract

Retinal degenerative diseases are one of the important refractory ophthalmic diseases, featured with apoptosis of photoreceptor cells. Histone acetylation and deacetylation can regulate chromosome assembly, gene transcription, and posttranslational modification, which are regulated by histone acetyltransferases (HATs) and histone deacetylases (HDACs), respectively. The histone deacetylase inhibitors (HDACis) have the ability to cause hyperacetylation of histone and nonhistone proteins, resulting in a variety of effects on cell proliferation, differentiation, anti-inflammation, and anti-apoptosis. Several HDACis have been approved for clinical trials to treat cancer. Studies have shown that HDACis have neuroprotective effects in nervous system damage. In this paper, we will summarize the neuroprotective effects of common HDACis in retinal degenerative diseases and make a prospect to the applications of HDACis in the treatment of retinal degenerative diseases in the future.

## 1. Introduction

A nucleosome is the fundamental unit of eukaryotic chromosomes, whereas the core of the nucleosome is composed of histones (H2A, H2B, H3, and H4). Histone acetylation and deacetylation can regulate the binding of DNA and transcription complexes and further regulate chromosome assembly, gene expression, mitosis, and posttranslational modification [1, 2]. Histone acetylation and deacetylation are regulated by histone acetyltransferases (HATs) and histone deacetylases (HDACs), respectively. HATs and HDACs can regulate the dynamic acetylation equilibrium of histone and nonhistone proteins and play an important role in cell proliferation, apoptosis, differentiation, angiogenesis, cancer treatment, neuroprotection, and anti-inflammatory effects [2, 3].

The histone deacetylase inhibitor (HDACi) can interfere with the deacetylase function of HDACs, improve the acetylation level of histone and nonhistone proteins, and regulate gene transcription. Clinically, HDACis are effective drugs in the treatment of a variety of cancers, such as pancreatic, ovarian, breast, colon, prostate, and thyroid cancer [4–9]. Large amounts of data have shown that HDACis also have important neuroprotective effects in the treatment of diseases of the nervous system [10–13]. HDACis are known to reduce apoptosis, increase cell survival, regulate the expression of various neurotrophic factors, and enhance anti-inflammatory responses [10, 11, 14–16]. Apoptosis of retinal photoreceptor cells is a main feature of retinal degenerative diseases [17, 18], and neurotrophic factors have positive protective effects on retinal degenerative diseases [19, 20]. Thus, HDACis may have therapeutic potentials for retinal degenerative diseases. In this paper, we will focus on the progress of studies on using HDACis in the prevention and treatment of retinal degeneration.

## 2. Histone Deacetylase

There are 18 HDACs in human, and they are divided into four different classes based on their homology to yeast protein RPD3, Hda1, Sir2, and HOS3 (Table 1) [3]. Classes I, II, and IV HDACs are Zn^2+^-dependent and homologous to the yeast RPD3, Hda1, and HOS3, respectively, whereas Class III HDACs are NAD^+^-dependent and homologous to yeast Sir2. Class I HDACs include HDACs 1, 2, 3, and 8, which are localized in the nucleus [21]. Class I HDACs can regulate neurogenesis, cell senescence, proliferation, differentiation, and embryonic development [22–25]. HDACs 4, 5, 6, 7, 9, and 10 make up Class II HDACs, which are localized both in nucleus and in cytoplasm. Class II HDACs consist of two subclasses: Class IIa (HDACs 4, 5, 7, and 9) and Class IIb (HDACs 6 and 10). Compared to Class I HDACs, Class II has more tissue-specific functions, such as cardiac, microtubule, and chondrocyte differentiation defects [26–28]. Class III HADCs consist of sirtuins (SIRT1–7), whereas Class IV contains only HDAC11 and relatively little is studied about this subtype [3, 21]. In this paper, we introduce mainly the progress of Class I and II HDACs inhibitors in the treatment of retinal degenerative diseases.

## 3. Histone Deacetylase Inhibitor

According to the different chemical structures, HDACis can be divided into four classes, which include hydroxamic acids, cyclic peptides, benzamides, and aliphatic acids [21, 29] (Table 2). Hydroxamic acids can inhibit Class I and Class II HDACs, which include trichostatin A (TSA), vorinostat (SAHA), panobinostat (LBH589), and belinostat (PXD101) [30–33]. Cyclic peptides, romidepsin (FK228), have the most complex structure. Benzamides include entinostat (MS-275) and mocetinostat (MGCD0103). Common aliphatic acids include valproic acid (VPA), sodium butyrate (NaB), and phenylbutyrate (PBA) [34]. HDACis can cause hyperacetylation of histone and nonhistone proteins and further regulate transcription process, cellular microenvironment, and immune responses [35]. HDACis have an important role in the inhibition of tumor cell proliferation and in the induction of cell differentiation [36–38]. Studies have shown that HDACis can promote the transcription of retinal photoreceptor genes by histone acetylation, resulting in effectively reversing the course of retinal photoreceptor cell degeneration [39–41]. Several HDACis have been approved for clinical trials, such as SAHA, FK228, Mgcd0103, LBH589, PXD-101, and MS-275 [35]. Currently, the studies of HDACis focus mainly on cancer therapy, cell differentiation, neuroprotection, and heterochromatin fields, and as yet, research has just started in retinal degeneration.

## 4. Trichostatin A

TsA is a hydroxamic acid, a Class I and II HDACi, which is the first natural hydroxamic acid found to inhibit HDACs, and is one of the most studied HDACis, especially in the retina [31]. TsA has an important role in the prevention and treatment of neurodegenerative conditions [12, 42]. TsA can regulate the levels of apoptosis-related proteins and improve neurological performance in the rat permanent middle cerebral artery occlusion (pMCAO) model of stoke [11] (Table 3).

TsA suppressed TNF-*α* expression and signaling in retina from rat ischemic injury and changed the level of acetylated histone 3 (AcH3) and the secretion of matrix metalloproteinase-1 (MMP-1) and MMP-3 [43]. TsA also improved the electroretinography (ERG) responses in ischemic injury retina [43, 44]. In the zebrafish retina, TsA can regulate cell-cycle progression and neurogenesis by Wnt and notch signaling pathways [22]. TsA also regulates the apoptotic process by upregulating the expression of apoptotic protease activating factor-1 (apaf-1) and caspase 3 in the developing mouse retina [41]. TsA treatment attenuated the downregulation of* Fem1c* ^*R3*^ gene expression, delayed the progressive damage, and reduced apoptosis to retinal ganglion cells (RGCs) in aged DBA/2J mice [45]. TsA induced axonal regeneration by inducting expression of AcH3 and retinoic acid receptor *β* (RAR*β*) in adult rat RGCs [46], which play an important role in development and differentiation [47]. In* in vitro* retinal explants of retinal degeneration 1 (*rd1*) mice, TsA treatment decreased the rate of cells apoptosis, enhanced the photoreceptor cell survival, and prevented photoreceptor degeneration by suppressing poly(ADP-ribose) polymerase (PARP) activity, which promoted cell death of* rd1 *retina [39, 48, 49]. However, in retinal explants of normal mice, TsA inhibited the expression of pro-rod transcription factors Otx2, Nrl, and Crx and the development of rod photoreceptor cells [40], which had the opposite effect compared with retinal degeneration mice. TsA treatment inhibited the proliferation and the TGF-*β*2-induced epithelial-mesenchymal transition (EMT) pathway by downregulating TGF-*β*/Akt, MAPK, ERK1/2, and notch signaling pathways in human retinal pigment epithelial (RPE) cells. This may have a clinical value in the prevention and treatment of proliferative vitreoretinopathy (PVR) [50] (Table 4).

## 5. Valproic Acid

As a short chain fatty acid, VPA is a broad-spectrum HDACi and is currently used widely as an anticonvulsant drug. Many studies have shown that VPA has neuroprotective effects against the damage of central nervous system (Table 3). VPA has been shown to reduce brain damage in a rat transient focal cerebral ischemia model and to improve functional outcome by reducing caspase 3 activation and increasing heat-shock protein 70 (Hsp70) levels [10]. In a rat pMCAO stoke model, VPA increased the anti-inflammatory effect by inhibiting inducible nitric-oxide synthase (iNOS) and OX-42, regulated the levels of apoptosis-related proteins, and improved neurological performance [11]. In rat intracerebral hemorrhage (ICH) model, VPA reduced perihematomal cell death and activities of caspases 3, 8, and 9 and alleviated inflammation by regulating transcriptional activation [51]. Under hypoxic conditions, VPA treatment prevented neuron apoptosis, increased levels of AcH3, activated NF-*κ*B, and reduced JNK activation in the primary rat hippocampal and cortical cultures* in vitro* [52].

VPA has also an important role in protecting the RGCs (Table 4). In a rat ischemia/reperfusion (I/R) model, VPA prevented axon damage of RGCs [14, 53]. After I/R damage, VPA attenuated retinal neuron apoptosis by inhibiting the activation of caspase 3, upregulation of apaf-1, and release of cytochrome C. At the transcriptional level, VPA upregulated the expression of Hsp70 and enhanced acetylation of histone H3 and Hsp70 promoter [14]. VPA treatment prevented significantly the retinal histological damage and the loss of RGCs by reducing endoplasmic reticulum (ER) stress-induced apoptosis. VPA decreased the expression of C/EBP homologous protein (CHOP) and caspase 12 [53]. CHOP is a transcription factor involved in ER stress-induced apoptosis [54], whereas caspase 12 is a proapoptotic factor activated by ER stress [55]. After optic nerve crush (ONC) in rat, VPA has a neuroprotective effect by increasing RGCs survival and expression of pERK1/2, inhibiting caspase 3 activity, and inducing the DNA binding of cAMP response element binding protein (CREB) in the injured RGCs [56]. In purified rat RGCs, VPA enhanced cell survival and delayed spontaneous cell death [57]. In a rat model of ONC, VPA treatment can inhibit apoptosis of RGCs via the activation of brain-derived neurotrophic factor (BDNF) and tropomyosin-related kinase B (TrkB) signaling [58]. VPA can induce expression of HSP70 and attenuate the photoreceptor cell death induced by N-methyl-N-nitrosourea in mice [59]. In clinical trials of retinitis pigmentosa (RP), VPA may reduce the loss of photoreceptor cells. VPA has an effective therapeutic potential for RP, but efficacy and safety of VPA in the treatment of RP need to be assessed by further clinical trials [60].

## 6. Sodium Butyrate

Sodium butyrate (NaB) is a short chain fatty acid, which can increase histone acetylation levels, inhibit tumor cell proliferation, and promote tumor cell senescence and apoptosis [61–64]. NaB is widely used as an animal feed additive [65] and plays also an important role in the prevention and treatment of neurodegenerative conditions [12, 13] (Table 3). It has anti-inflammatory effects in rat brain-derived primary microglia cells [66]. In the ischemic brain of pMCAO rat, NaB stimulated neurogenesis and induced cell proliferation, migration, and differentiation by BDNF-TrkB signaling [15]. Like VPA, NaB also has anti-inflammatory effects and neuroprotective effects in the rat pMCAO stroke model [11]. NaB can induce the activation of BDNF promoter IV in the rat cortical neurons* in vitro* [16]. NaB can regulate G1-to-S cell cycle progression by cyclin-dependant kinase (cdk) inhibitors p21 and p27 in adult mouse neural stem cells (NSCs) [67].

*In vitro*, NaB can delay spontaneous cell death, enhance cell survival in purified rat RGCs, and increase levels of AcH3 and AcH4 [57]; it can also increase the level of AcH3 and induce morphological changes in Y79 cells, a retinoblastoma cell line [68]. After NaB treatment, original round morphology of Y79 cells changed into spindle or irregular morphology. After ONC injury in rat, NaB can promote survival of RGCs, increase ERG responses, upregulate phosphorylation of Akt and Erk, and increase hyperacetylation of histone H3K14 [58] (Table 4).

## 7. Other HDACis 

SAHA, a hydroxamic acid derivative, is the first HDACi drug approved by the Food and Drug Administration (FDA) for the treatment of cancer in the United States [21]. In clinical trials, SAHA has been used to treat cutaneous T-cell lymphoma. Many studies have also shown that SAHA has neuroprotective effects [69–72]. Like NaB, SAHA can also regulate cell cycle progression by p21 and p27 in adult mouse NSCs [67] and SAHA also has a good protective effect in corneal haze and injury [73, 74]. SAHA can induce caspase-dependent apoptosis and reduce cell survival in human retinoblastoma (RB) cells [75, 76]. MS-275, a synthetic benzamide derivative, which selectively inhibits HDACs 1, 2, and 3, is also a HDACi drug used in cancer treatment in clinical trials. Ms-275 can protect RGCs differentiation and survival following optic nerve injury in Thy-1 CFP mice [77].

## 8. Discussion

Retinal degenerative diseases, such as RP, Leber congenital amaurosis (LCA2), achromatopsia, juvenile macular degeneration, and cone-rod dystrophy, are the major blinding fundus diseases, and the pathogenesis of these diseases is very complex. Apoptosis of photoreceptor cells is a common feature of retinal degeneration, and a variety of stimuli, such as tumor necrosis factor (TNF), Fas ligands (FasL), mitochondria, and ER stress, can lead to cell death. These stimuli can cause caspase cascade, activate firstly the initiator caspases (caspase 8, 9, 10, and 12), further activate downstream effector caspases (caspase 3, 6, and 7), and lead to apoptotic cell death [55], whereas antiapoptotic HSP70, B-cell lymphoma-2 (Bcl-2), and B-cell lymphoma-extra large (Bcl-xL) can inhibit this caspase cascade [11, 55]. HDACis can upregulate the expression of antiapoptotic HSP70 and Bcl-2 and downregulate the expression of proinflammatory TNF-*α* [11, 78, 79]. In retinal diseases, studies showed that HDACis treatment upregulated the expression of Hsp70, downregulated the expression of apaf-1 and caspase 3, inhibited the translocation of cytochrome C and activation of Akt and Erk, increased the rate of cell survival, and decreased the apoptosis process [14, 49, 58]. Akt and Erk signaling can inhibit apoptosis by preventing cytochrome C release [55]. VPA, NaB, and TsA regulate the activation of Akt and Erk signaling and further regulate the apoptosis process [50, 58].

Some factors, such as growth factors and cytokines, can activate PI3K/Akt, PKC, and Erk signaling, prevent the expression of antiapoptotic glycogen synthase kinase-3 (GSK-3), forkhead in rhabdomyosarcoma (FKHR), Bcl-2 antagonist of cell death (Bad), and Bcl-xL, and increase cell survival [55]. Neurotrophic factors also regulate the apoptosis of photoreceptor cells in the development of the visual system [55]. Ciliary neurotrophic factor (CNTF) can control photoreceptor differentiation in rat retina [80]. HDACis, VPA, NaB, and TSA increased the expression of glial cell line-derived neurotrophic factor (GDNF) and BDNF in the rat astrocytes [81]. In* rd1* retinal explants, BDNF and CNTF activate the Erk, Akt, and CREB pathways to decrease the apoptosis of photoreceptor cells [82]. After ONC in rat, HDACis activate BDNF-TrkB signaling, upregulate the level of antiapoptotic Bcl-2, and downregulate the activation of caspase 3 [58]. These data suggest that HDACis have the potential to alter gene expression of neurotrophic factors and further regulate the apoptosis of photoreceptor cells in the retina.

Gene regulation is also an important function of HDACis in retinal degenerative diseases. Since the acetylation/deacetylation of histone and nonhistone proteins has extensive effects on gene regulation, upregulation of acetylation caused by HDACis would likely lead to significantly altered transcription of genes related to retinal degeneration. HDACis have been shown to inhibit the expression of FasL and proinflammatory cytokine interleukin-6 (IL-6), increase the acetylation of histone H3, activate the transcription of downstream genes Akt, Erk, CREB, and HSP70, and thus unregulated the levels of antiapoptotic proteins Bcl-2 and Bcl-xL, and eventually lead to the downregulation of caspase 3 [11, 51]. In retinal degenerative diseases, HDACis treatment can induce acetylation of histone H3, regulate Akt, Erk, CREB, and TrkB signaling, and further inhibit the activity of caspase 3 [14, 56, 58]. HDACis also can regulate the expression of neurotrophic factors [58].

Several factors can lead to the death of photoreceptor cells. In addition to spontaneous apoptosis and retinal degeneration, certain ocular adverse events, such as surgery and gene therapy, can also lead to the loss of photoreceptor cells. Gene therapy has broad application prospects and has achieved great success in the treatment of LCA2 [83]. It has been reported that gene therapy can restore visual function in animal models and clinical trials; but apoptosis of remaining photoreceptor cells could progress slowly and continuously in treated areas, and the restored visual function by gene therapy gradually weakens [83–86]. In addition to retinal detachment caused by subretinal injections and the release of toxic substances around the treated areas, continued photoreceptor loss is also related to photoreceptor cells having begun the irreversible apoptosis process before treatment [84, 87]. It is important to correct the negative effects of gene therapy that appeared in the ongoing clinical trial, and HDACis may be a good option. Considering the fact that HDACis can prevent death of photoreceptor cells and protect retinal damage, we hypothesize that HDACis may play a role in preventing the continuing death of photoreceptor cells after gene therapy and are conducting these experiments.

**Figure 1:**
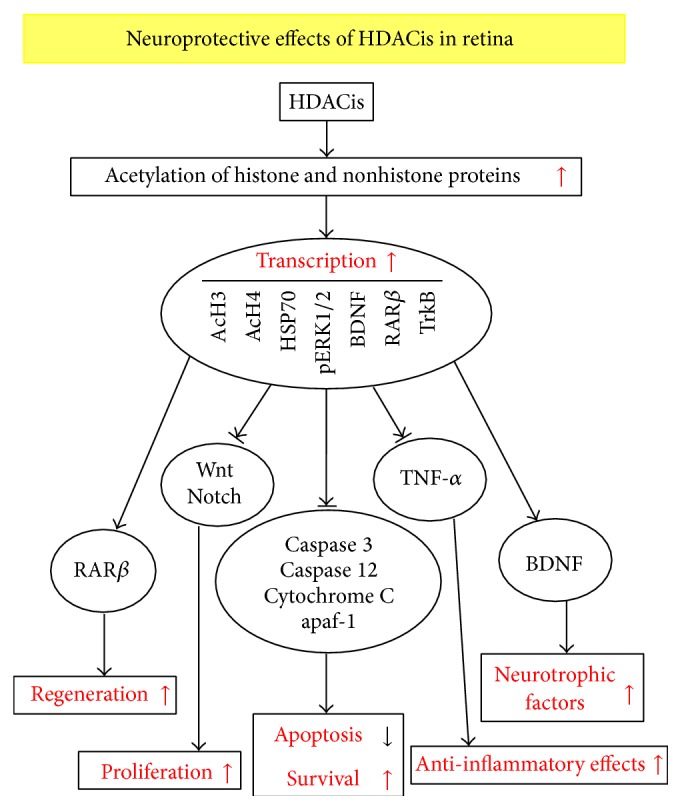
Possible mechanisms of HDACis in the prevention of retinal degenerative diseases. HDACis acetylate histone and nonhistone proteins, such as AcH3, AcH4, and HSP70, regulate transcription process. HDACis promote cell regeneration and proliferation, improve cell survival, enhance anti-inflammatory effects, attenuate cell apoptosis, and upregulate the expression of neurotrophic factors.

In this paper, we summarized the neuroprotective effects of common HDACis in retinal degenerative diseases (Figure 1). Currently, clinical trials of VPA in RP have been carried out. As in-depth studies of HDACis, more and more molecular mechanisms of HDACis on neuroprotective effects will be found in retinal degenerative diseases. HDACis can inhibit the apoptosis of photoreceptor cells during retinal damage process; therefore, HDACis may be a group of promising agents to be explored in the prevention of apoptosis of photoreceptors and in the treatment of retinal degenerative diseases.

## Figures and Tables

**Table 1:** Class, homology, catalytic subunit, compound, and localization of HDACs.

Class	Homology	Catalytic subunit	Compound	Localization	References
I	RPD3	Zn^2+^	HDACs 1–3 and 8	Nucleus	[3, 21]
IIa	Hda1	Zn^2+^	HDACs 4, 5, 7, and 9	Nucleus/cytoplasm	[3, 21]
IIb	Hda1	Zn^2+^	HDACs 6 and 10	Mostly cytoplasm	[3, 21]
III	Sir2	NAD^+^	SIRT1–7	Nucleus/cytoplasm	[3, 21]
IV	HOS3	Zn^2+^	HDAC 11	Nucleus/cytoplasm	[3, 21]

**Table 2:** Class, common compound, HDAC target, and main functions of HDACis.

Class	Compound	HDAC target	Function	References
Hydroxamic acids	TsA SAHA LBH589 PXD101	Classes I and II Classes I and II Classes I and II Classes I and II	A, D, GA, P, CP, R, NG, and AI A, CP, S, TR, and AI A, GA, TR, and P A, GA, and TR	[21, 22, 41, 42, 88] [21, 67, 69, 70, 74] [21, 33, 89] [21, 33]

Cyclic peptides	FK228	Class I	A, GA, D, and TR	[21, 33, 90]

Benzamides	MS-275 MGCD0103	HDACs 1, 2, and 3 Class I	A, D, S, and GA A, TR, AI, and GA	[21, 77] [21, 91, 92]

Aliphatic acids	VPA PBA NaB	Classes I and IIa Classes I and IIa Classes I and IIa	A, AI, TR, S, D, and GA A, D, and GA A, D, GA, AI, TR, S, and NG	[10, 11, 21, 51] [21, 93, 94] [11, 15, 21, 57]

A: cell apoptosis/death; AI: anti-inflammatory effect; TR: transcriptional regulation; NG: neurogenesis; S: cell survival; CP: cell-cycle progression; P: proliferation; R: regeneration; D: differentiation; GA: growth arrest.

**Table 3:** Function and molecular targets of common HDACis in nervous system diseases.

HDACi	Function	Molecular targets	References
TSA	A AI TR	Bcl-2 and apaf-1 IL-6, TNF-*α*, and NF-kappaB HSP70, AcH3, AcH4, PI3K/Akt, BDNF, and NF-*κ*B	[11] [42] [11]

VPA	A AI TR	Caspase 3 and HSP70 OX-42, ED-1, and iNOS HSP70, AcH3, pERK, bcl-2, pCREB, pAkt, bcl-xl, NF-*κ*B, and JNK	[10, 11, 51, 52] [11] [10, 11, 51, 52]

NaB	AI N A TR	OX-42, ED-1, and iNOS BDNF-TrkB Caspase 3 and HSP70 HSP70, AcH3, AcH4, Sp1, p21, and p27	[11] [16] [11] [11, 67]

A: cell apoptosis/death; AI: anti-inflammatory effect; TR: transcriptional regulation; N: neurogenesis.

**Table 4:** Function and molecular targets of common HDACis in retinal degenerative diseases.

HDACi	Function	Molecular targets	References
TsA	CP P A R AI TR	Wnt signaling and notch signaling Notch signaling, cyclinD1, CDK, and p-Rb Caspase 3, apaf-1, and PARP RAR*β* and AcH3K9 TNF-*α* AcH3, TNF-*α*, MMP-1, and MMP-3	[22] [22] [41, 49] [46] [43] [43]

VPA	A S TR	Caspase 3, Caspase 12, apaf-1, HSP70, and cytochrome C Caspase 3, CREB, and pERK1/2 HSP70, AcH3, cytochrome C, GRP78, CHOP, TrkB, and pERK1/2	[14, 56, 58, 59] [14, 56, 58, 59] [14, 56, 58, 59]

NaB	A S TR	BDNF-TrkB and AcH3K14 AcH3 and AcH4 AcH3, AcH4, Akt, and Erk	[58] [57] [57]

A: cell apoptosis/death; AI: anti-inflammatory effect; TR: transcriptional regulation; S: cell survival; CP: cell-cycle progression; P: proliferation; R: regeneration.
